# Paradoxical sparing of cerebral circulation in massive TAVR-associated endocarditis

**DOI:** 10.1186/s13019-026-04338-9

**Published:** 2026-06-15

**Authors:** Kang An, Marc Gillinov, Lars G. Svensson, Miralem Pasic

**Affiliations:** 1 Department of Thoracic and Cardiovascular Surgery Cleveland, Cleveland Clinic, Cleveland, OH USA; 2 Department of Thoracic and Cardiovascular Surgery, Heart, Vascular & Thoracic Institute, Desk J4-1, 9500 Euclid Ave, Cleveland, 44195 OH USA

**Keywords:** Transcatheter aortic valve replacement, Prosthetic valve endocarditis, Systemic emboli

## Abstract

**Background:**

Prosthetic valve endocarditis (PVE) after transcatheter aortic valve replacement (TAVR) is an uncommon but life-threatening complication associated with high embolic risk.

**Case presentation:**

A 82-year-old woman with prior TAVR presented with fever and malaise and was diagnosed with PVE due to Streptococcus Mutans. Transesophageal echocardiography revealed a large, highly mobile vegetation attached to the transcatheter valve, floating in the systolic jet of blood flow. Computed tomography demonstrated progressive splenic infarction and embolic involvement of the kidneys and mesenteric circulation, while no cerebral embolization occurred. At surgery, the previously visualized vegetation was no longer present, suggesting interval embolization. The transcatheter valve was explanted using cardiopulmonary bypass and cardioplegia. Radical debridement and annular enlargement with bovine pericardium were performed, followed by implantation of a surgical bioprosthesis using infection-conscious techniques. The patient recovered uneventfully. Post hoc analysis may suggest that proximal origin of the supra-aortic vessels (type III aortic arch configuration with the innominate artery originates below the horizontal plane of the inner curvature of the aortic arch) in the setting of an elongated aorta in this patient may explain the absence of cerebral embolization. This could possibly be relevant in combination with the centrally located floating vegetation, which was ejected along the strong central flow stream and consequently embolized to the abdominal organs. However, this excludes a generalization of the statement, and therefore the classic surgical principle of early surgery in patients with huge floating vegetations should be applied in similar cases.

**Conclusions:**

This case illustrates the embolic potential of large, mobile vegetations in TAVR-associated endocarditis and supports early surgical intervention in accordance with both American and European infective endocarditis guidelines. The absence of cerebral embolization in the presence of massive systemic emboli should not be mistaken for anatomical protection.

**Supplementary Information:**

The online version contains supplementary material available at 10.1186/s13019-026-04338-9.

## Background

Prosthetic valve endocarditis (PVE) after transcatheter aortic valve replacement (TAVR) is an uncommon but life-threatening complication. The incidence is approximately 3% among TAVR patients [1]. Targeted antibiotic treatment is important for PVE. However, there are certain situations where medical management alone has not been enough. Both American College of Cardiology/American Heart Association guidelines and European Society of Cardiology guidelines recommend early surgery in infective endocarditis with large mobile vegetations, systemic embolization, or prosthetic valve involvement [2, 3]. Here we report a case of PVE complicated by multiple septic emboli but paradoxically sparing the cerebral circulation, which required an early surgical intervention.

## Case presentation

An 82-year-old women presented to the local hospital with intermittent fever, malaise, and fatigue for three months. She was diagnosed with PVE and transferred to our center for the surgical management. On admission, she was hemodynamically stable with blood pressure 155/78mmHg, sinus rhythm at 101 beats/min, oxygen saturation of 98%, and temperature 36.6℃.

### Past medical history

The patient underwent TAVR in August 2024 for severe aortic stenosis (26-mm Sapien 3 valve). She had acute pulmonary embolism in April 2024, which was treated with three months of direct oral anticoagulants. Additional history included asthma, hypertension, and hyperlipidemia.

### Investigations

**Fig. 1:**
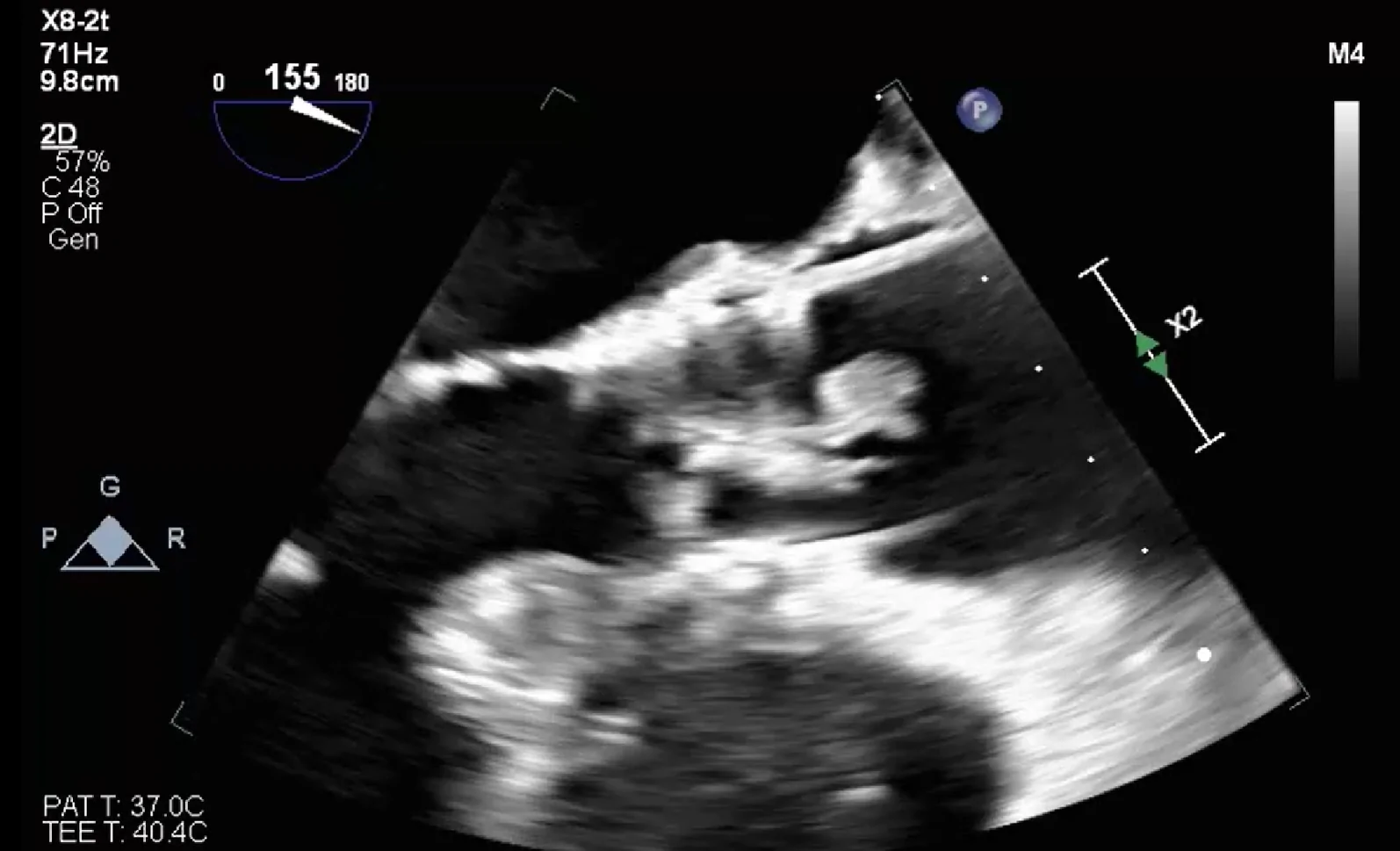
Preoperative transesophageal echocardiography demonstrated a large (about 25 mm), elongated, highly mobile vegetation attached to the transcatheter aortic valve prosthesis

At the referring hospital:

- Blood cultures grew Streptococcus mutans
- Transthoracic echocardiogram (TTE) showed elevated trans-prosthetic gradients (The peak gradient was 54 mmHg and the mean gradient was 32 mmHg. There is no aortic valve regurgitation.)
- Transesophageal echocardiogram (TEE) revealed a large, elongated, highly mobile vegetation (about 25 mm) attached to the prosthetic valve, floating in the systolic jet (Fig. 1 and Video 1)
- Computed tomography (CT) showed no cerebral embolization, but computed tomography angiography (CTA) showed a large splenic infarction (5–6 cm), later progressing to involve nearly 80% of the spleen and of both kidneys and mesenteric circulation. Also positive for acute pulmonary emboli within the bilateral lower lobe segmental pulmonary artery
- A 2.8cm polypoid filling defect was found in the cecum/ascending colon, suspicious for developing neoplasm

**Fig. 2:**
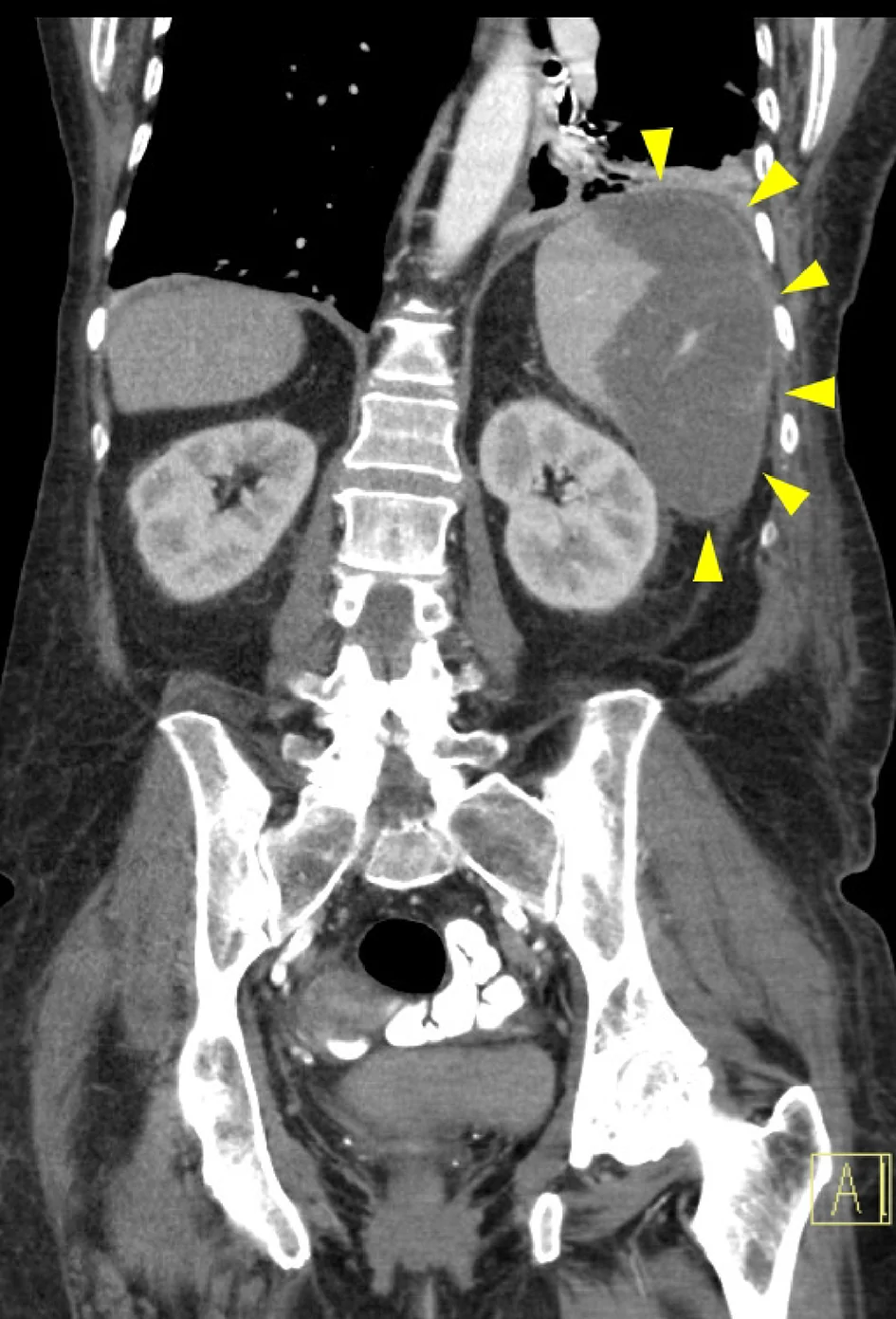
Computed tomography angiography showed extensive splenic infarction with near-complete involvement of the splenic parenchyma (arrowheads)

**Fig. 3:**
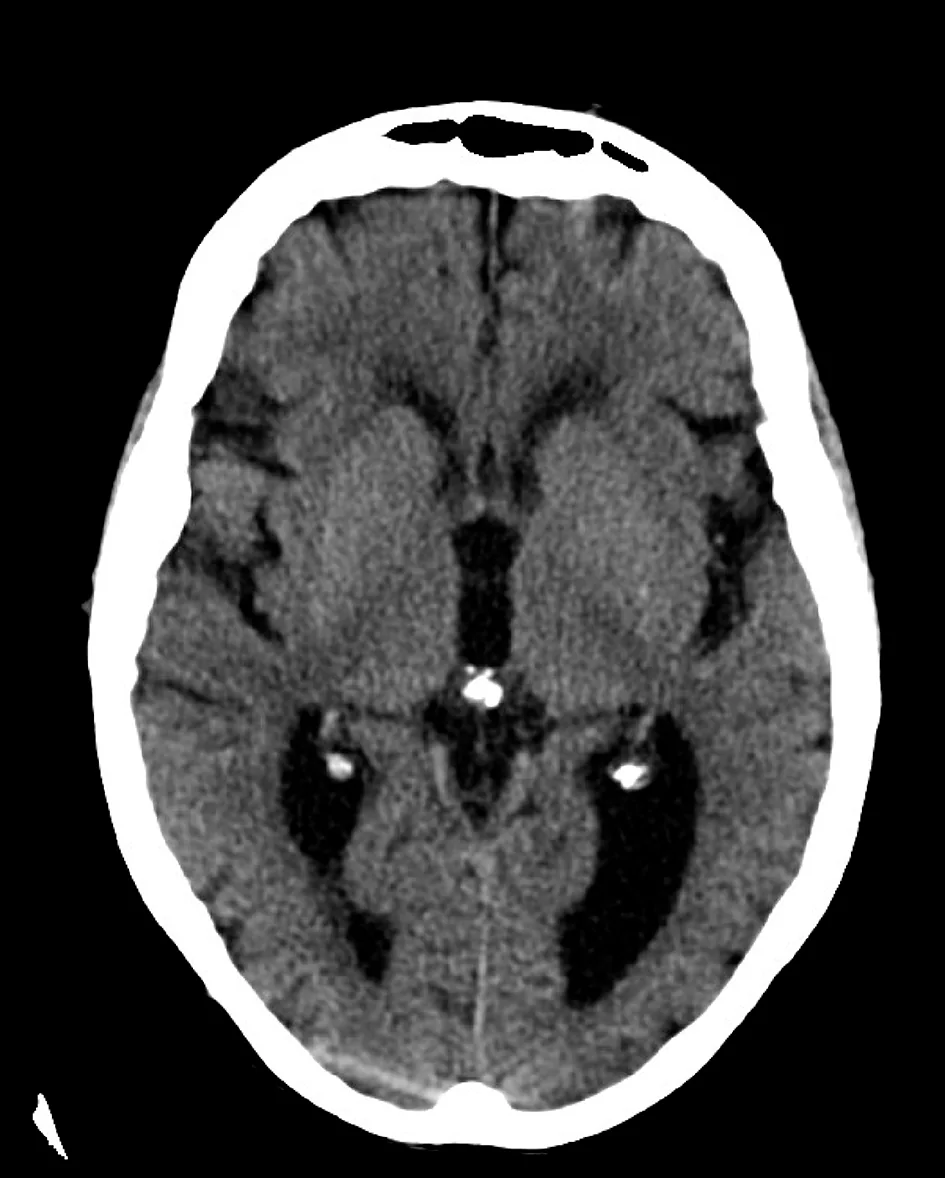
Computed tomography of head revealed no cerebral embolism

After transferred to our center:

- Repeat TTE suggested persistent prosthetic valve infection
- Repeat CTA confirmed progression of splenic infarction (Fig. 2)
- Repeat CT did not reveal cerebral embolization (Fig. 3)

### Differential diagnosis

Differential diagnoses included structural valve degeneration and tumor fever associated with possible colorectal cancer. Progressive embolization and positive blood cultures confirmed infective endocarditis.

## Management

### Medical management

Broad-spectrum intravenous antibiotics were initiated and later tailored to culture results. Splenectomy was deferred as no abscess was present.

### Surgical treatment

Intraoperative TEE did not find large, mobile vegetation seen in the previous TEE (Video 2). The transcatheter valve was explanted during conventional surgery using cardiopulmonary bypass and cardioplegia. We employed standard TAVR explant technique [4, 5]. The aorta is opened via a nearly vertical incision extending from the upper left toward the lower right, reaching the edge of the TAVR prosthesis in the region of the non-coronary sinus. Subsequently, using a vascular dissector, the endothelialized portion of the stent of the Sapien valve is very precisely separated from the aortic wall. Once this part of the stent is detached from the wall of the aorta, the vertical incision is extended into the non-coronary sinus of Valsalva.

**Fig. 4:**
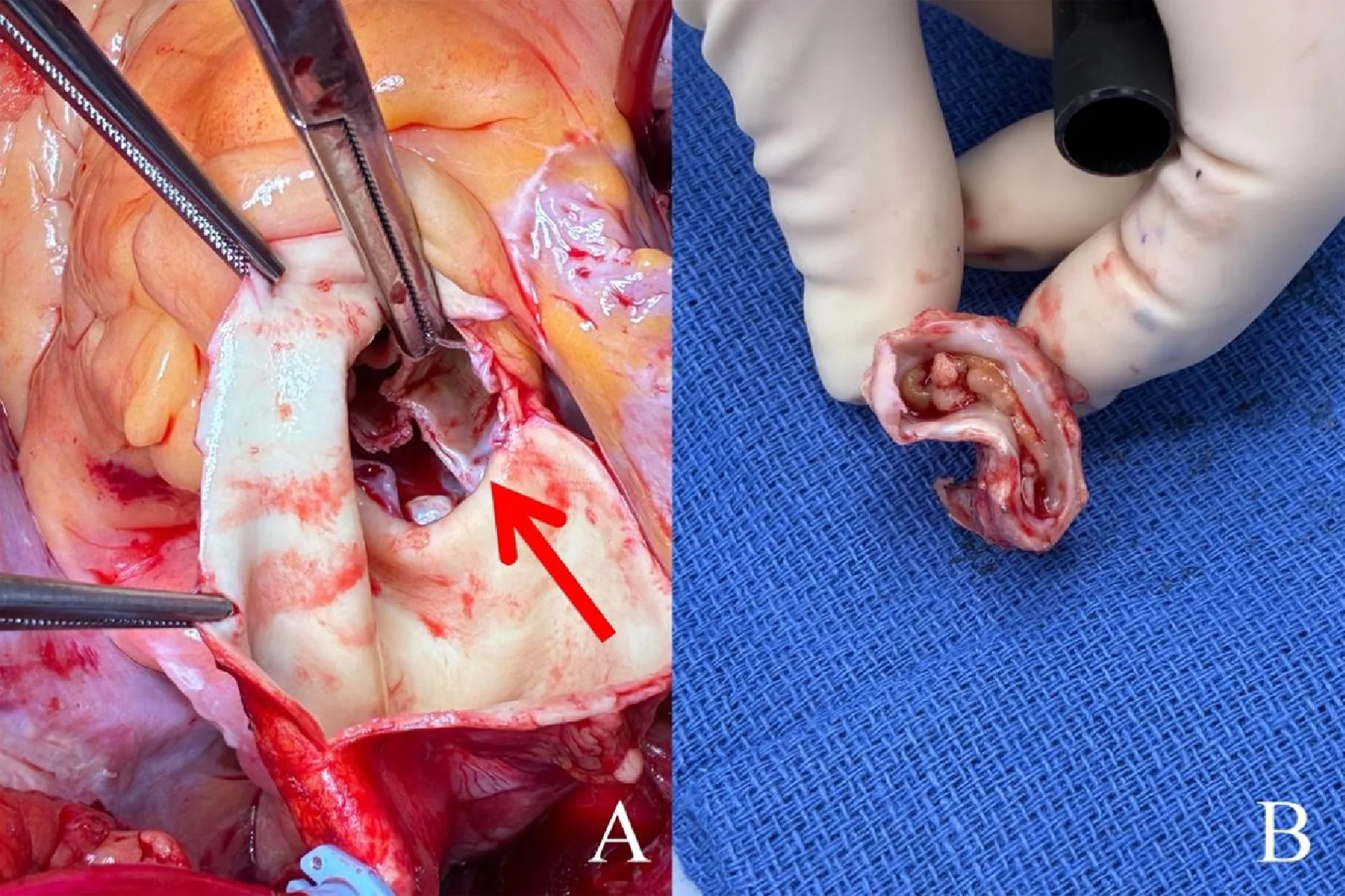
Intraoperative Findings.(**A**) No large adherent vegetation was found on the prosthesis(**B**) Ventricular side of the explanted balloon-expandable transcatheter heart valve (Sapien 3) showed small vegetations. The valve deformation occurred as a result of squeezing during the explantation

The second phase involves using two sturdy instruments (for example, a Pean or Kocher clamp) to grasp opposite sides of the prosthesis. These are simultaneously pulled toward the center of the prosthesis to deform it, which opens the plane between the Sapien prosthesis and the native valve leaflets. Care must be taken during this step to avoid damaging any vegetations, preventing the risk of subsequent embolism. The deformed Sapien prosthesis is then further separated from the native leaflets using a dissector, which is now a straightforward process. Finally, the native leaflets are excised in the classic manner, and the operation proceeds. The large vegetation was not found intraoperatively (Fig. 4), strongly suggesting embolization. Radical debridement of infected tissue was performed. To implant the largest possible valve in this patient with a gracile body habitus (body mass index: 24.6 kg/m^2^; body surface area: 1.59 m), the small aortic annulus was enlarged. A bovine pericardial patch (Edwards Lifesciences, Irvine, CA, USA) was chosen for its superior resistance to infection compared to fabric patches and was tailored accordingly to fit the anatomy. An Inspiris RESILIA aortic valve size 23 (Edwards Lifesciences, Irvine, CA, USA) was implanted. The valve was implanted using modified techniques to reduce prosthetic material burden, including Prolene 2 − 0 U-sutures supported with bovine pledgets. Prior to implantation, the prosthetic sewing ring was soaked in vancomycin solution. The procedure was completed without complications.

### Outcome and follow-up

**Fig. 5:**
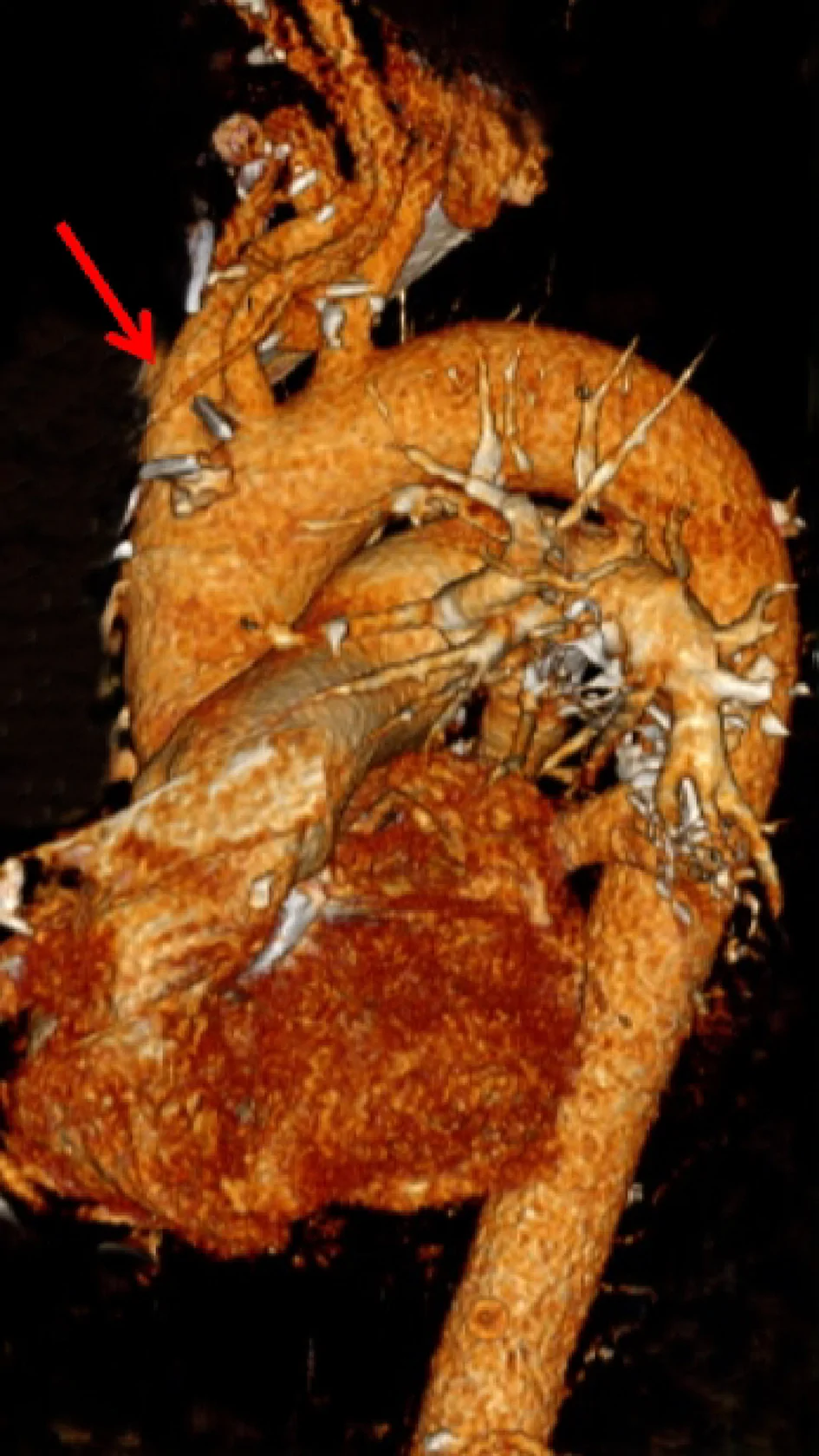
Computed tomography angiography revealed an elongated aorta and a proximal origin of the supra-aortic vessels. As a result, the main flow jet is not directed toward the orifices of the supra-aortic vessels

The patient had an uneventful recovery. She was extubated shortly after surgery and discharged. Her outpatient plan including additional six weeks of intravenous ceftriaxone and anticoagulation with apixaban. No neurological complications occurred. Postoperative CTA reconstruction revealed a proximal origin of the supra-aortic vessels, suggesting a direct anatomical path (Fig. 5).

## Discussion and conclusions

This case uniquely documents a long, floating vegetation visualized preoperatively that disappeared before surgery, correlating with progressive embolic infarctions in multiple organs (spleen, kidneys, mesentery), while sparing the brain. This represents a hemodynamic paradox where the proximal displacement of the supra-aortic branches might serve as a protective mechanism. The high-velocity jet originating from the TAVR prosthesis created a linear momentum that projected the large vegetation beyond the proximal orifices—a phenomenon known as embolic overshooting [6–8]. Combined with centrifugal forces directing the mass toward the ‘empty’ greater curvature of the arch, the embolic material was effectively shunted into the descending aorta, sparing the cerebral circulation.

When analyzing the anatomy of the aortic arch and the origins of the supra-aortic vessels, it is essential to consider the classification into Type I, II, and III configurations [9]. In our case, the patient’s anatomy corresponded to a Type III aortic arch, characterized by the innominate artery originating below the horizontal plane of the inner curvature of the arch. Literature suggests that a Type III configuration generates a unique, abnormal helical flow pattern, which has been identified as a potential risk factor for type B aortic dissection due to its specific hemodynamic profile [10]. We propose that this same hemodynamic environment played a decisive role in our patient; the specific helical flow and linear momentum likely redirected the embolic material away from the supra-aortic orifices. This pattern facilitated the ‘embolic overshooting’ of the large vegetation, effectively shunting it into the descending aorta and resulting in the paradoxical sparing of the cerebral circulation. It is also remarkable that despite multiple systemic emboli and a large vegetation visualized prior to surgery but not found on surgery, the brain remained unaffected on both occasions. This raises the question of whether this repeated preservation of the brain was sheer coincidence or the result of a specific hemodynamic pattern.

The “embolic overshooting” hypothesis explains the absence of stroke through fluid dynamics (linear momentum and centrifugal forces) rather than random chance. It suggests that a large mass can “miss” the supra-aortic vessels because of its velocity and the specific anatomy of the aortic arch. Nevertheless, “luck” may be a more robust explanation for several reasons. Primarily, vegetation consistency: vegetations are usually friable, breaking into smaller pieces that lack the mass to overshoot and are instead swept into the head vessels. Additionally, diastolic flow: the significant turbulence and retrograde flow during diastole can move a floating mass toward the arch vessels regardless of its systolic momentum. Therefore, embolic overshooting remains a possible case-specific mechanism but lacks consistency to be a general clinical rule.

However, it should be emphasize that this theoretical considerations are not a stable or reliable protective mechanism. A more proximal origin of the supra-aortic vessels does not in itself create a mechanical barrier that protects the brain. The current absence of cerebral emboli most likely reflects the present flow distribution and the characteristics of the fragments detaching from the vegetation, rather than anatomical protection. It should be noted that large vegetations are typically friable and prone to fragmentation, generating smaller emboli that can easily enter the high-flow supra-aortic branches. Moreover, aortic flow is not confined to systole; diastolic flow reversal and turbulence within the aortic arch might promote embolic migration toward the supra-aortic vessels. In the setting of a large, mobile vegetation on a TAVR prosthesis with already documented multiple systemic emboli, the brain must still be considered a high-risk territory — it simply has not yet been in the dominant path of embolic flow. Therefore, embolic overshooting should be interpreted as a case-specific mechanism rather than a broadly applicable clinical rule.

About 15–20% of patients with PVE after TAVR received surgical treatment, compared with 50% of patients with PVE after surgical aortic valve replacement [11, 12]. Surgical treatment of PVE TAVR is challenging. Infection-conscious strategies such as radical debridement, use of biologic materials, annular enlargement, and minimizing foreign material may contribute to improved outcomes.

In conclusion, this case highlights the embolic danger of large, floating vegetations in TAVR-associated endocarditis. Early surgery, guided by multimodality imaging and performed with infection-adapted surgical techniques, can be lifesaving and is supported by contemporary American and European guidelines. The absence of cerebral embolization in the presence of massive systemic emboli should not be mistaken for anatomical protection.

## Electronic Supplementary Material

Below is the link to the electronic supplementary material.

Supplementary material 1:Video 1. Initial transesophageal echocardiography showed a long, highly mobile vegetation attached to the transcatheter valve frame, oscillating freely and floating in the systolic blood flow jet, consistent with high embolic potential.

Supplementary material 2: Video 2. Intraoperative transesophageal echocardiography did not find the previously seen vegetation.

## Data Availability

No datasets were generated or analysed during the current study.

## Abbreviations

CT: Computed tomography
CTA: Computed tomography angiography
PVE: Prosthetic valve endocarditis
TAVR: Transcatheter aortic valve replacement
TEE: Transesophageal echocardiography
TTE: Transthoracic echocardiography
